# Raster plots machine learning to predict the seizure liability of drugs and to identify drugs

**DOI:** 10.1038/s41598-022-05697-8

**Published:** 2022-02-10

**Authors:** N. Matsuda, A. Odawara, K. Kinoshita, A. Okamura, T. Shirakawa, I. Suzuki

**Affiliations:** 1 Department of Electronics, Graduate School of Engineering, Tohoku Institute of Technology, 35-1 Yagiyama Kasumicho, Taihaku-ku, Sendai, Miyagi 982-8577 Japan; 2 Drug Safety Research Labs, Astellas Pharma Inc., 21 Miyukigaoka, Tsukuba, Ibaraki 305-8585 Japan

**Keywords:** Biological techniques, Biophysics, Biotechnology, Drug discovery, Neuroscience

## Abstract

In vitro microelectrode array (MEA) assessment using human induced pluripotent stem cell (iPSC)-derived neurons holds promise as a method of seizure and toxicity evaluation. However, there are still issues surrounding the analysis methods used to predict seizure and toxicity liability as well as drug mechanisms of action. In the present study, we developed an artificial intelligence (AI) capable of predicting the seizure liability of drugs and identifying drugs using deep learning based on raster plots of neural network activity. The seizure liability prediction AI had a prediction accuracy of 98.4% for the drugs used to train it, classifying them correctly based on their responses as either seizure-causing compounds or seizure-free compounds. The AI also made concentration-dependent judgments of the seizure liability of drugs that it was not trained on. In addition, the drug identification AI implemented using the leave-one-sample-out scheme could distinguish among 13 seizure-causing compounds as well as seizure-free compound responses, with a mean accuracy of 99.9 ± 0.1% for all drugs. These AI prediction models are able to identify seizure liability concentration-dependence, rank the level of seizure liability based on the seizure liability probability, and identify the mechanism of the action of compounds. This holds promise for the future of in vitro MEA assessment as a powerful, high-accuracy new seizure liability prediction method.

## Introduction

In the field of drug discovery and development, one major factor leading to delays and failures in the development of new drugs is the cancellation of development because of side effects discovered in nonclinical studies and clinical trials^1^. Compounds that display central nervous system (CNS) toxicity constitute 34% of all candidate compounds that are removed from candidacy in the clinical stage^2^. Detection of CNS toxicity in preclinical trials is an important topic of study in modern drug development^3^. Drug-induced seizures have a severe CNS side effect; the most common method of in vitro evaluation of seizures is a rat hippocampal brain slice assay^4^. Brain slice assays use tissue from the brain of an animal, meaning that the slice has a functional network of neurons, making it the in vitro gold standard^5^. However, the slices deteriorate quickly after they are harvested; hence, in general, a brain slice assay requires sample preparation and experiments to be conducted on the same day; one issue with this is that the throughput thus yielded is low.

One high-throughput in vitro functional evaluation method that uses cultured neurons involves performing measurements with a planar microelectrode array (MEA). Use of an MEA is a noninvasive method of measuring neural network activity at multiple points simultaneously^6–13^. With modern advancements in multiwell testing, the effects that compounds have on nerve function can be measured in a high-throughput fashion^14,15^. This holds promise as an in vitro seizure toxicity evaluation method^16,17^. Previous studies have used rodent neurons to detect seizure-causing compound response^18–20^.

Because conventional brain slice assays and MEA assays use rodent cells, they lack human receptors and other potential drug targets, creating issues with interspecies extrapolation. The use of human induced pluripotent stem cell (iPSC)-derived neurons^21,22^ holds promise as a method that can guarantee the ability to extrapolate data to humans. In recent years, many researchers, including our research group, have published research on neurotoxicity screening via MEA using human iPS cells^13,15,23–31^.

However, the analytic methods and analytic parameters for the detection of seizure toxicity in in vitro MEA assay are not well established^16,17^. Seizure-causing compounds with different mechanisms have different end point changes^18,20,23^. In other words, using only simple end points for general seizure-like events (SLEs) such as the number of network bursts and firing activity is insufficient; there is a need to develop multiple end points and new analytic parameters.

Multiple end points obtained from MEA data are generally calculated by plotting the neural network activity time-series data as a raster plot^14,18,19,23,27,28,31,32^. Many parameters are related to network bursts^11,18,20,23,26,28,31^, but the methods for detecting network bursts differ between facilities^11,18,33^, and there is a problem that the differences in the methods for detecting network bursts affect the results^33^. Raster plots contain the all spike information of a neural network activity, but sufficient information on the neural network activity inherent in the spike column cannot be extracted from the conventional analysis parameters. Therefore, herein, we propose an analysis method that uses deep learning to extract 4096-dimensional features from raster plot images and machine-learn the features. This is because detecting network bursts is not necessary, and there is a possibility of capturing changes in neural network activity from the information of all spike sequences.

Thus, machine learning using raster plots is considered an effective method of toxicity screening. Previously published research on seizure detection via machine learning includes methods of SLEs identification using detection in electrocorticograms after seizures^34^, rat acute slice local field potential signals, and image recognition technology^35^; however, there are no previous publications on the use of raster plots for machine learning.

In the present study, we obtained MEA data from 13 seizure-causing compounds and two seizure-free compounds using human iPSC-derived cortical neurons and developed a method for distinguishing between seizure-causing compounds and seizure-free compounds as well as identifying drugs using machine learning trained on raster plots.

## Results

### Human iPSC-derived neural network drug response

**Figure 1:**
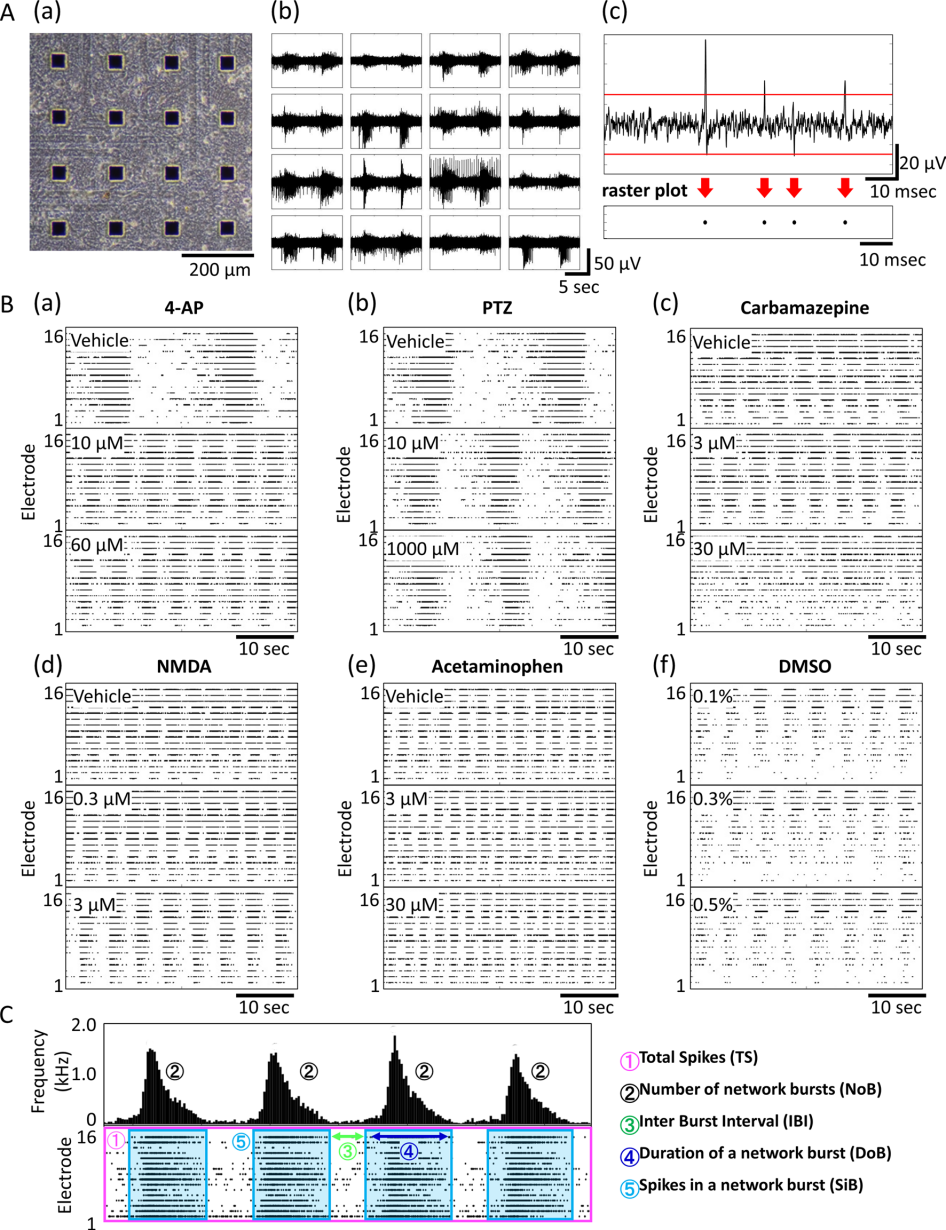
MEA data from a cultured human iPSC-derived neural network. **(A) (a)** Phase-contrast image of neurons on an MEA chip at 81 days in vitro (DIV). **(b)** Typical action potential waveform in a spontaneous recording. **(c)** Upper graph shows the action potential waveform acquired with a single electrode and the voltage threshold for spike detection (red line). Raster plots of detected spikes (black circles) are shown under the graph. **(B)** Concentration-dependent Raster plot images of typical mechanisms of action **(a)** 4-AP, **(b)** carbamazepine, **(c)** NMDA, **(d)** PTZ, **(e)** acetaminophen, **(f)** DMSO. **(C)** Schematic diagram of analysis parameters.

**Figure 2:**
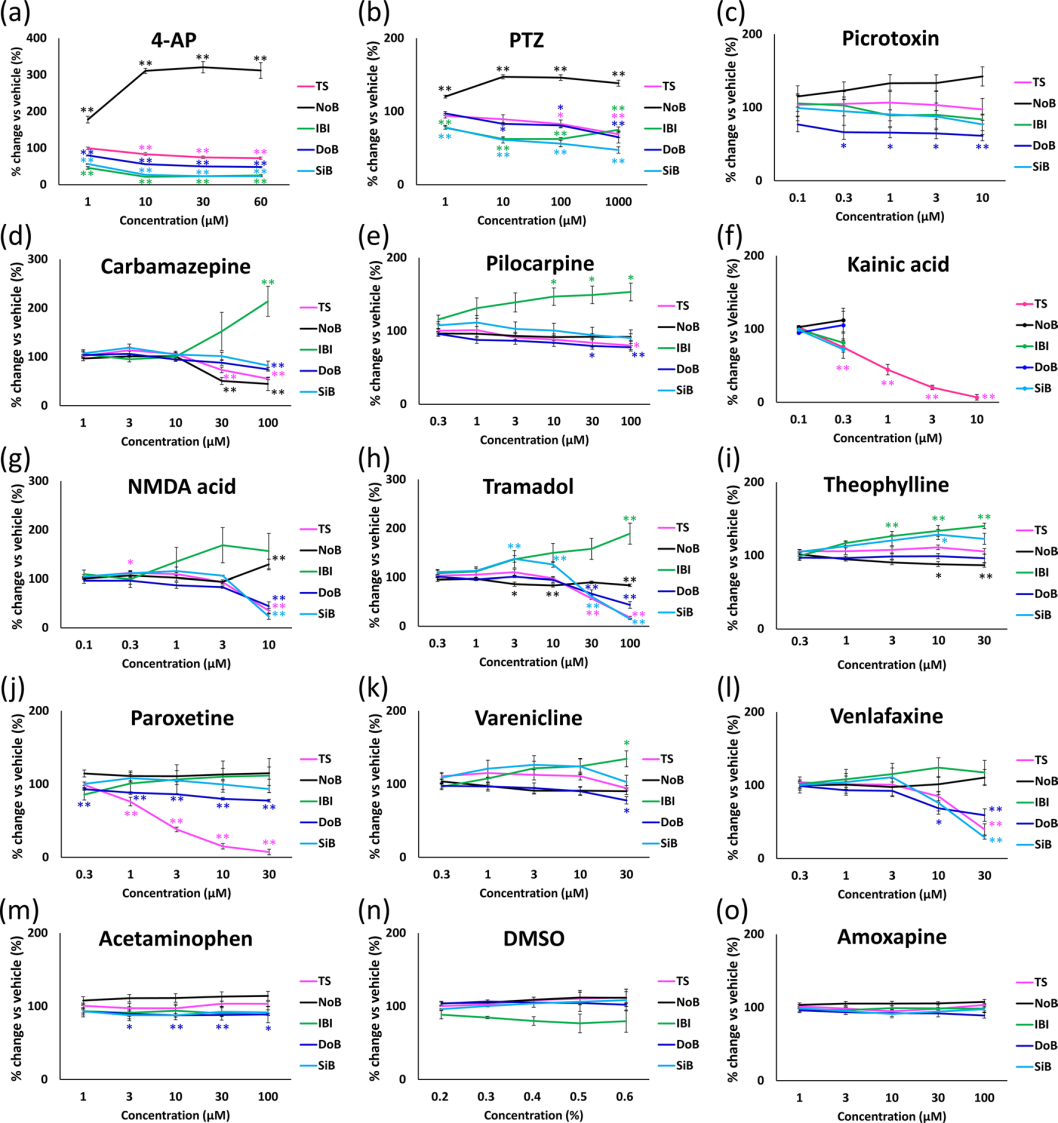
Concentration-dependent changes of 15 compounds in five parameters: TS (pink), NoB (black), IBI (green), DoB (blue), SiB (cyan). Parameters were depicted as the average % change of control (vehicle control set to 100%) ± SEM from n = 3–4 wells. Data were analyzed using one-way ANOVA followed by post hoc Dunnett's test (*p < 0.05, **p < 0.01 vs. vehicle).

Culturing of a human iPSC-derived neural network seeded on an MEA was possible without cell aggregation even on the 12th week of culturing. Network burst firing was observed from the 6th week of culture onward. Figure 1A (a) shows a cultured 81 days in vitro (DIV) phase contrast image, and Fig. 1A (b) shows a typical network burst signal. Concentration-dependent data were obtained for 13 seizure-causing compounds and two seizure-free compounds after the 14th week of culturing, when the neural networks were considered mature^28^. Whenever the signal that was obtained passed a threshold, the spikes detected were used to create a raster plot. Figure 1A (c) shows the threshold used to detect spikes in the single electrode signal (top portion) and a raster plot of the detected spikes (bottom portion). Figure 1B shows raster plots of compounds with different mechanisms of action: (a) 4-aminopyridine (4-AP), (b) pentylenetetrazol (PTZ), (c) carbamazepine, (d) N-methyl-D-aspartic acid (NMDA), (e) acetaminophen, and (f) dimethyl sulfoxide (DMSO). Seizure-causing compounds caused different changes depending on their mechanism of action (Fig. 1B). Figure 1C shows a schematic of five analytic parameters calculated from raster plots (total spikes (TS), number of network bursts (NoB), inter network burst interval (IBI), duration of a network burst (DoB), and spikes in a network burst (SiB)). Figure 2 shows the drug response of each parameter when the vehicle response is set to 100%. The numerical data are listed in supplementary Tables S1–S5. The maximum increases in the NoB of 4-AP and PTZ were 321.0% ± 15.4% (30 µM) and 147.3% ± 2.7% (10 µM), respectively. The IBI, DoB, and SiB decreased starting at a concentration of 1 µM for 4-AP and PTZ (Fig. 2a,b). The DoB decreased starting at 0.3 µM of picrotoxin (Fig. 2c). For carbamazepine, the TS and NoB decreased at 30 µM, and the DoB decreased and the IBI increased at 100 µM (Fig. 2d). For pilocarpine, the IBI increased starting at 10 µM, the DoB decreased starting at 30 µM, and the TS decreased at 100 µM (Fig. 2e). For kainic acid, the TS decreased at 0.3 µM and the NoB went to 0 starting at 1 µM (Fig. 2f). For NMDA, the TS increased at 1 µM whereas the TS, DoB, and SiB decreased and the NoB increased at 10 µM (Fig. 2g). For tramadol, the NoB decreased and the SiB increased starting at 3 µM, the TS, DoB, and SiB decreased at 30 µM, and the IBI increased at 100 µM (Fig. 2h). For theophylline, the IBI increased starting at 10 µM, and the SiB increased whereas the NoB decreased starting at 30 µM (Fig. 2i). For paroxetine, the DoB decreased starting at 0.3 µM, and the TS decreased starting at 1 µM (Fig. 2j). For varenicline, the IBI increased and the DoB decreased at 30 µM (Fig. 2k). For venlafaxine, the DoB decreased at 10 µM, and the TS and SiB decreased at 30 µM (Fig. 2l). For acetaminophen, the DoB decreased starting at 3 µM (Fig. 2m). For DMSO and amoxapine, no changes in any parameters were observed (Fig. 2n,o).

Based on the preceding results, we found that the changes in the parameters studied were not similar among all seizure-causing compounds; changes differed based on the mechanism of action of the drug. At the same time, a significant difference in the DoB was detected for acetaminophen, which is a seizure-free compound. Changes in DoB may be observed for certain seizure-free compounds. Consequently, we found that there are difficulties in using a single parameter to distinguish between seizure-causing compounds with different mechanisms of action and seizure-free compounds.

### Creating an artificial intelligence using raster plots and evaluating drugs as either seizure-causing or seizure-free

**Figure 3:**
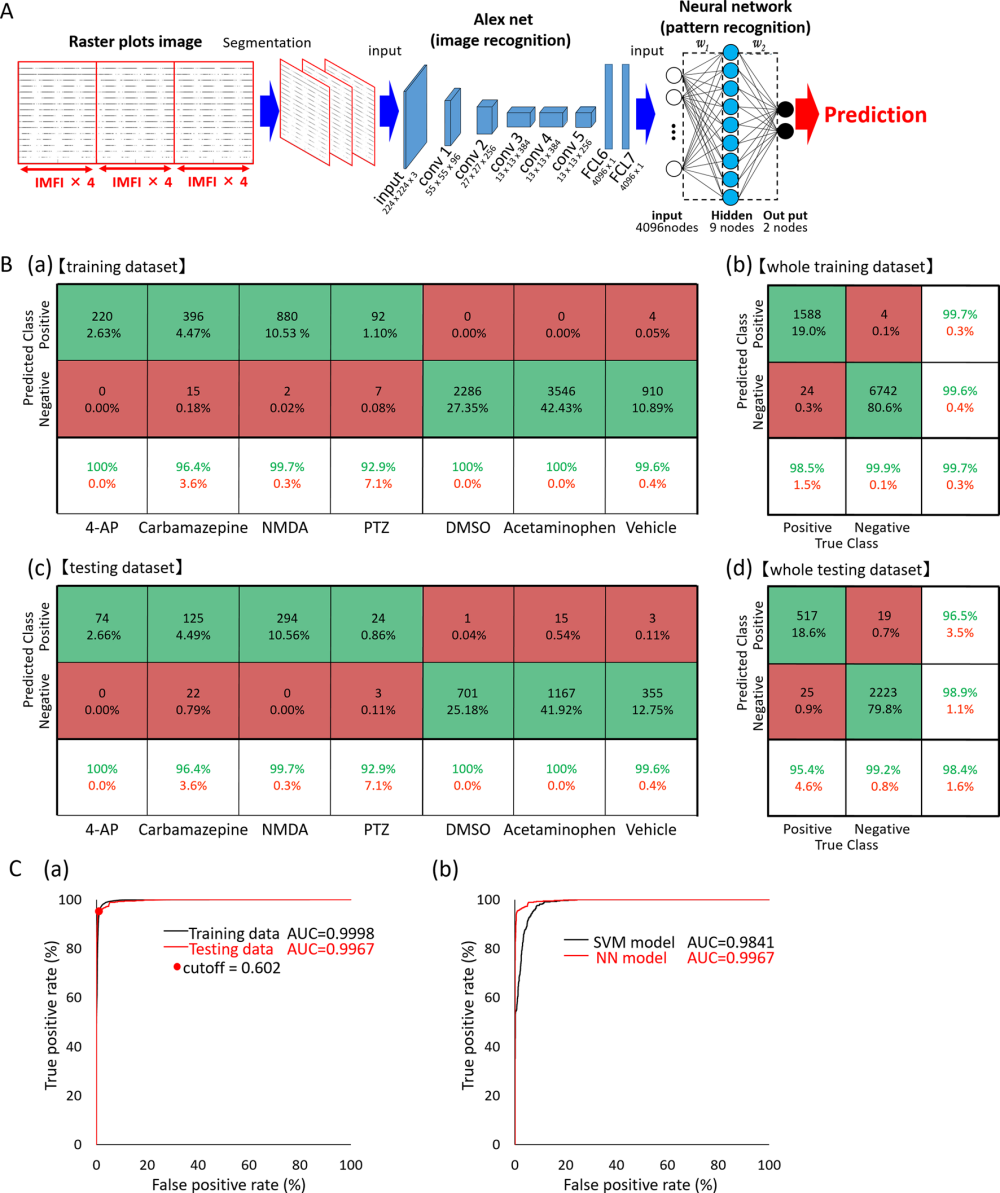
Creation of seizure risk prediction AI using raster plot images and evaluation of the prediction model. **(A)** Data flow and architecture of seizure risk prediction model. w1 is the weight between the input layer and the hidden layer, w2 is the weight between the hidden layer and the output layer. **(B)** **(a)** Confusion matrix for each compound used for training, **(b)** confusion matrix for the entire training dataset, **(c)** confusion matrix for each compound used for the test, **(d)** confusion matrix for the entire test dataset. The test dataset used the data of the wells not used for training dataset. Vehicle in the confusion matrix indicates vehicle data in four seizure-causing compounds. **(C)** **(a)** Receiver operating characteristic (ROC) curve after classification of training and testing data in a neural network model (black line: training data; red line: testing data; red dot: optimum operating point). **(b)** Comparison of ROC curves after classification of the same testing data in NN and SVM models (black line: SVM model; red line: NN model).

We created an artificial intelligence (AI) that was trained on raster plots so that it could classify the responses of seizure-causing compounds with different mechanisms of action as well as the responses of seizure-free compounds. Raster plots were created from the time-series data of the detected spikes, and then images were created by segmenting the data into time windows four times that of the inter-maximum frequency of a network burst interval (IMFI) in the pre-drug administration. The network burst frequency differed depending on the well, so the number of segmented raster plot images also differed depending on the well. The reason for choosing four times the IMFI is that it is suitable for capturing both the regularity of network burst activity and fine firing patterns, and reduces variability between wells. Next, the segmented raster plot images were input into AlexNet^36^, an object recognition model, and the 4096-dimensional parameters which were output from the fully connected layer (the 21st layer) were extracted as image feature quantities. Lastly, we corrected for differences between the wells due to differing initial states by normalizing the feature quantities of each drug around the mean value of the feature quantities when the vehicle was administered to each well. The 13 seizure-causing compound and two seizure-free compound datasets, which is the number of split raster plots per concentration, were created as shown in Table 1. We used a pattern recognition neural network composed of 4096 neuron input layers, nine sigmoid neuron hidden layers, and an output layer with two classes, which made up a toxicity prediction model to predict whether a compound was a seizure-causing compound or a seizure-free compound (Fig. 3A). We used four seizure-causing compounds with different mechanisms and burst frequency responses (4-AP [30 and 60 µM, n = 3 wells, respectively], carbamazepine [100 µM, n = 3 wells], NMDA [3 and 10 µM, n = 3 wells], and PTZ [1000 µM, n = 3 wells]) and two seizure-free compounds (all concentrations of acetaminophen [n = 3 wells] and all concentrations of DMSO [n = 3 wells]) to train and validate the effectiveness of this model; 75% of the dataset was used for training, and the remaining 25% was used for validation after training (Table 1). The reason for selecting the four seizure-causing compounds is that, in order to cover the firing pattern of the seizure-causing compound, compounds having different mechanisms of action were selected as training data, one in which the firings increased and the other in which the firings decreased. The accuracy was evaluated using the raster plots of unlearned wells after training, i.e., using the holdout scheme. The training data used contained 330 4-AP plots, 822 carbamazepine plots, 1323 NMDA plots, 198 PTZ plots, and 3546 acetaminophen plots and DMSO 2286 plots. The test data used contained 111 4-AP plots, 294 carbamazepine plots, 441 NMDA plots, 54 PTZ plots, and 1182 acetaminophen plots and 702 DMSO plots. We created a confusion matrix of the seizure-causing and seizure-free classification results from the training data and test data (Fig. 3B). Next, a receiver operating characteristic curve and the area under the curve (AUC) were calculated for all training data and all test data, and the optimal operating point was determined (Fig. 3C(a)). The accuracy, positive predictive value, sensitivity, specificity, and F-measure of the prediction results of the model at the optimal operating point were calculated (Table 2). The model trained on raster plot feature quantities had an AUC in the training data of 0.9998 and an AUC in the unlearned data of 0.9967; the optimal operating point was 0.158. The classification precision in the training data for each drug at the optimal operating point was as follows: 100% for 4-AP, 97.8% for carbamazepine, 99.6% for NMDA, and 96.0% for PTZ. The classification precision in the unlearned data was 100% for 4-AP, 91.5% for carbamazepine, 100% for NMDA, and 94.4% for PTZ. The prediction accuracy for all compounds was 98.4%.

**Table 1:**
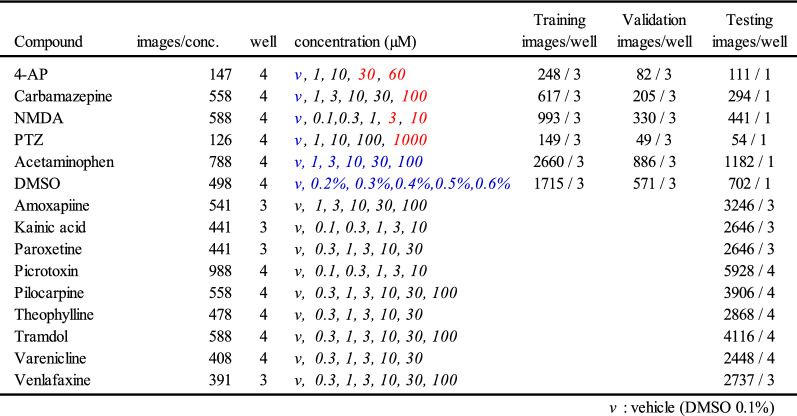
Dataset information using creation of seizure risk prediction AI.

The concentration in red letters indicates the concentration learned as seizure-causing, and blue letters indicate the concentration learned as seizure-free.

**Table 2:** Predictive model performance evaluation using the optimum operating point.

Original/predict	Training					Training				
	Acc	PPV	Sen	Spec	F-measure	Acc	PPV	Sen	Spec	F-measure
All compounds	0.997	0.997	0.985	0.999	0.991	0.984	0.965	0.954	0.992	0.959
4-AP	1.000	1.000	1.000	1.000	1.000	1.000	1.000	1.000	1.000	1.000
Carbamazepine	0.978	1.000	0.964	1.000	0.981	0.915	0.977	0.850	0.980	0.909
NMDA	0.996	0.997	0.998	0.993	0.997	1.000	1.000	1.000	1.000	1.000
PTZ	0.960	0.989	0.929	0.990	0.958	0.944	1.000	0.889	1.000	0.941

*Acc* accuracy, *PPV *positive predictive value, *Sen*  sensitivity, *Spec* specificity.

Figure 3C(b) shows the ROC curve using a support vector machine (SVM) model trained with the same 4096-dimensional feature dataset as the neural network (NN) model. Comparing the AUC in the test data of SVM and NN revealed that the NN model had an AUC of 0.9967 and the SVM model had an AUC of 0.9841; thus, the NN model was superior to the SVM model [Fig. 3C(b)]. Therefore, in this study, we used the NN model.

The seizure-causing/seizure-free classification AI trained on the raster plots that we created accurately classified the responses of seizure-causing compounds with differing mechanisms and seizure-free compounds.

### Determining concentration-dependent seizure toxicity using the AI

**Figure 4:**
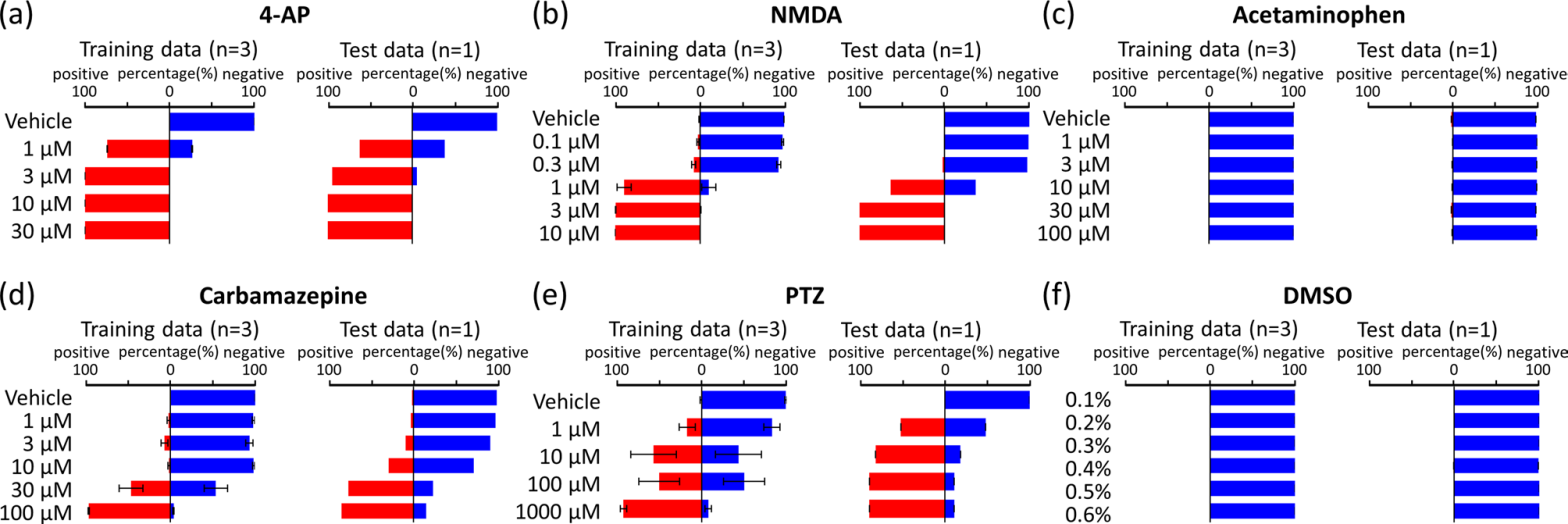
Concentration-dependent prediction of seizure risk in learning drugs by AI. AI predicted the negative probabilities (blue bar) and seizure risk (red bar) at each concentration of training data (left) and test data (right). **(a)** 4-AP, **(b)** NMDA, **(c)** acetaminophen, **(d)** carbamazepine, **(e)** PTZ, **(f)** DMSO.

If we are able to establish a ranked development priority for compounds based on their seizure liability, it will lead to more efficient drug discovery and development. Determining the concentration dependence is necessary in order to assign priority to drugs. Thus, using the AI we created, we investigated the concentration dependence of seizure-causing/seizure-free judgments. The concentration data toxicity probabilities predicted by the AI are shown in Fig. 4. The proportions of the images classified as seizure-causing and as seizure-free used the time-series data from each well, and then the mean probability for each well was calculated and used to represent the toxicity risk at each concentration. For unlearned sample, which includes data of the wells that were not used for training dataset, the following concentrations were determined to have a seizure liability probability of 50% or higher—4-AP: 1 µM (62.2%), 10 µM (94.6%), 30 µM (100%), and 60 µM (100%); carbamazepine: 30 µM (76.9%) and 100 µM (85.0%); NMDA: 1 µM (63.3%), 3 µM (100%), and 10 µM (100%); and PTZ: 1 µM (51.9%), 10 µM (81.5%), 100 µM (88.9%), and 1000 µM (88.9%) (Fig. 4 (a), (b), (d), (e)). The seizure liability at concentrations lower than the concentrations the AI was trained on was shown, and then the concentration dependence was calculated. Acetaminophen, which is a seizure-free compound, was determined to be seizure-free with a probability of 97.9% or higher, regardless of the concentration. DMSO was also determined to be seizure-free with a probability of 99.1% or higher, regardless of the concentration (Fig. 4c,f). The seizure liability prediction AI we created determined the concentration dependence of seizure-causing compounds and identified seizure-free compounds as seizure-free regardless of the concentration.

### Determining the seizure toxicity of unlearned drugs using the AI

**Figure 5:**
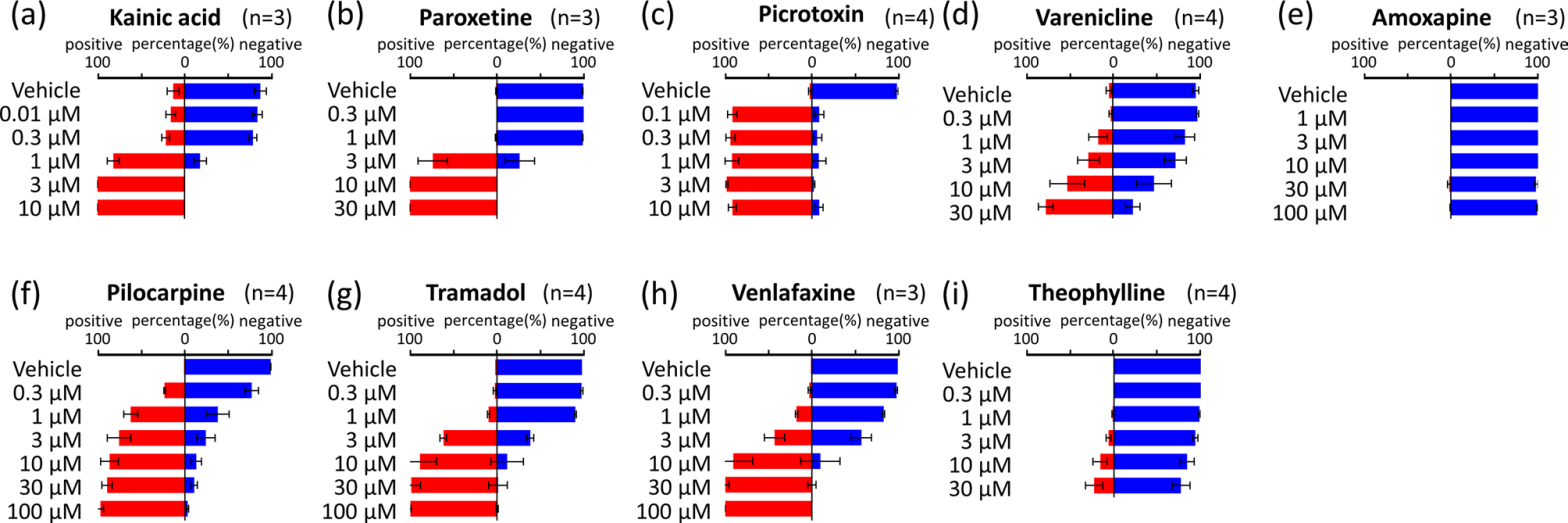
Concentration-dependent prediction of seizure risk in non-training drugs by AI. AI predicted the negative probabilities (blue bar) and seizure risk (red bar) at each concentration. **(a)** Kainic acid, **(b)** paroxetine, **(c)** picrotoxin, **(d)** varenicline, **(e)** amoxapine, **(f)** pilocarpine, **(g)** tramadol, **(h)** venlafaxine, **(i)** theophylline.

It is important for the AI that we created to detect the toxicity of drugs that it has not been trained on. Thus, we used the AI we created to determine the toxicity of nine unlearned seizure-causing compounds based on data collected on them. In order to verify AI, nine unlearned seizure-causing compounds were regarded as unknown compounds and were not trained. Figure 5 shows the seizure toxicity determination results for each concentration of the unlearned drugs. The concentrations that showed a 50% or higher probability of seizure liability were as follows—kainic acid: 1 µM (81.8%), 3 µM (100%), and 10 µM (100%); paroxetine: 3 µM (73.7%), 10 µM (100%), and 30 µM (100%); picrotoxin: 0.1 µM (91.4%), 0.3 µM (93.7%), 1 µM (91.8%), 3 µM (97.8%), and 10 µM (91.5%); varenicline: 10 µM (52.6%) and 30 µM (77.1%); pilocarpine: 1 µM (62.3%), 3 µM (75.8%), 10 µM (86.8%), 30 µM (89.4%), and 100 µM (97.0%); tramadol: 3 µM (61.9%), 10 µM (88.6%), 30 µM (98.9%), and 100 µM (100%); and venlafaxine: 10 µM (90.5%), 30 µM (100%), and 100 µM (100%). Seven of the unlearned drugs were determined to have concentration-dependent seizure liability (Fig. 5a–d, f–h). On the other hand, amoxapine and theophylline were determined to be seizure-free at all concentrations (Fig. 5e,i). This showed that the AI was able to detect seizure toxicity in a concentration-dependent manner, even for unlearned drugs.

**Figure 6:**
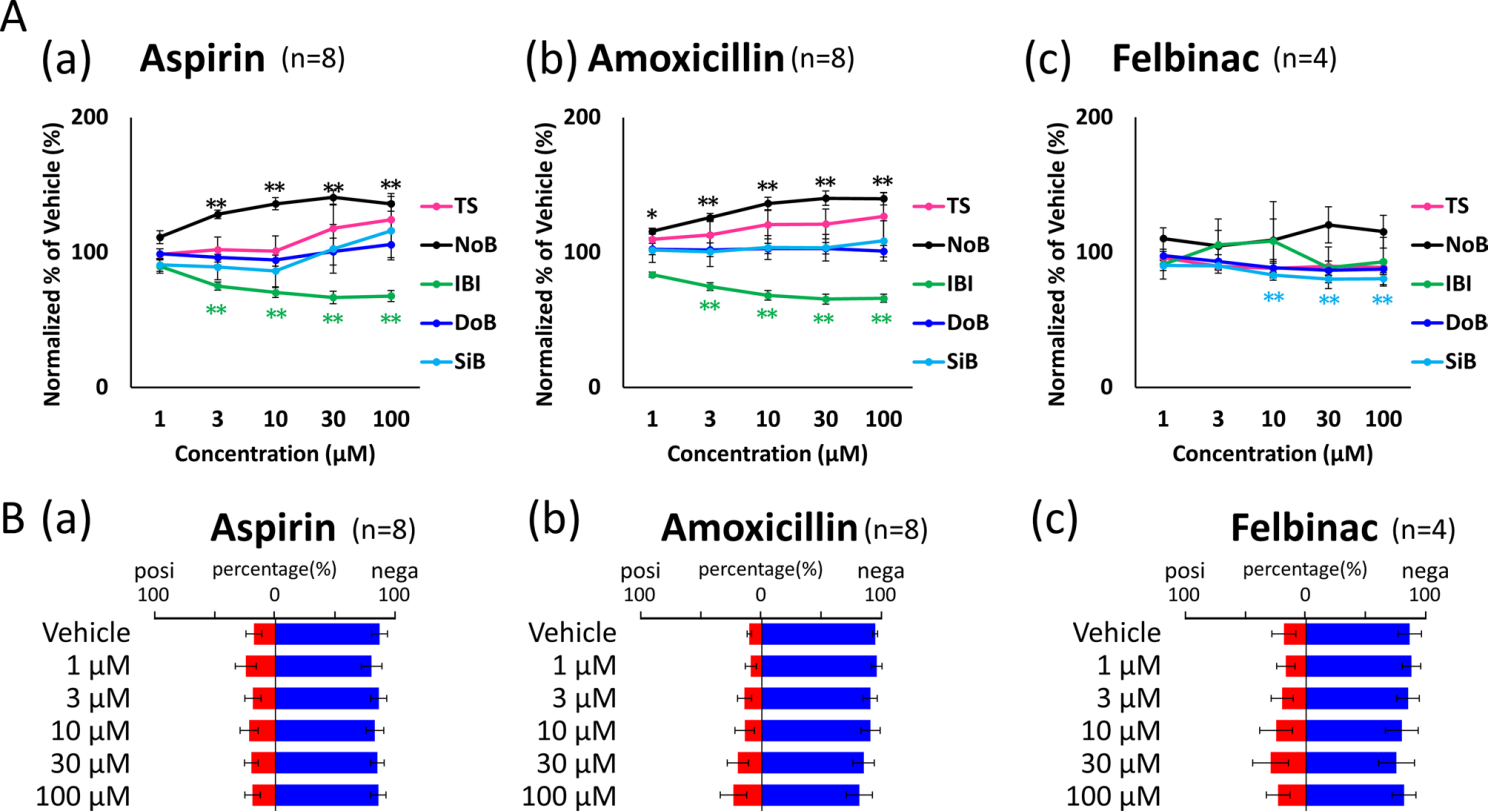
Prediction of seizure risk in non-training negative compounds by AI. **(A)** Concentration-dependent changes of 3 negative compounds in five parameters: TS (pink), NoB (black), IBI (green), DoB (blue), SiB (cyan). **(a)** Aspirin, **(b)** amoxicillin, **(c)** felbinac, **(B)** AI predicted the negative probabilities (blue bar) and seizure risk (red bar) at each concentration.

In order to verify whether AI can determine the safety of unlearned negative compounds, the negative compounds Aspirin (1, 3, 10, 30, 100 µM) and Amoxicillin (1, 3, 10, 30, 100) µM) and Felbinac (1, 3, 10, 30, 100 µM) data were judged (Fig. 6). The negative probabilities of Aspirin were 76.3% (1 µM), 82.0% (3 µM), 79.0% (10 µM), 80.8% (30 µM), and 81.7% (100 µM). Amoxicillin were 91.3% (1 µM), 86.3% (3 µM), 86.4% (10 µM), 81.1% (30 µM), and 77.6% (100 µM). Felbinac were 83.8% (1 µM), 80.9% (3 µM), 76.1% (10 µM), 71.8% (30 µM), and 77.7% (100 µM) (Fig. 6b). Although there were some significant differences in the conventional analysis parameters (Fig. 6a), AI judged negative at all three concentrations. From these results, it was confirmed that AI can be judged to be negative even for negative compounds that are unlearned drugs.

### Drug identification using the AI

Because seizure-causing compounds with differing mechanisms elicit different responses, if the AI is able to classify the compounds despite this, it can also predict the mechanism of seizure liability of unlearned drugs. Thus, we trained the AI on drug names and raster plots in order to classify compounds as seizure-causing compounds with differing mechanisms or seizure-free compounds.

**Figure 7:**
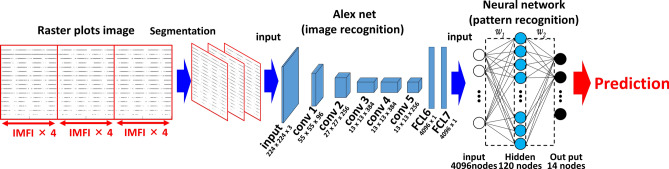
Creation of drug name prediction AI using raster plot images. Data flow and architecture of drug name prediction model. w1 is the weight between the input layer and the hidden layer; w2 is the weight between the hidden layer and the output layer.

We used a pattern recognition neural network composed of 4096 neuron input layers, 120 hidden layers containing sigmoid neurons, and an output layer with 14 classes (Fig. 7), which made up a drug identification model to predict the name of seizure-causing compounds and seizure-free compounds. The model was trained on a dataset composed of 4-AP (30 and 60 µM), amoxapine (100 µM), carbamazepine (30 and 100 µM), kainic acid (1, 3, and 10 µM), NMDA (3 and 10 µM), PTZ (1000 µM), paroxetine (3, 10, and 30 µM), picrotoxin (1, 3, and 10 µM), pilocarpine (10, 30, and 100 µM), theophylline (100 µM), tramadol (30 and 100 µM), varenicline (30 µM), and venlafaxine (10, 30, and 100 µM) as well as all concentrations of acetaminophen as well as all concentrations of DMSO as seizure-free compounds (Table 3). The all compounds dataset that was used was made up of 56 wells. Training was conducted by excluding one of the 56 wells and training the AI on the names of the drugs in the other 55 wells; 75% of the 55 well datasets were used for training, and 25% were used for validation after training. The excluded well was used for obtaining test data. The prediction accuracy was calculated using the leave-one-sample (well)-out scheme. We created five AIs for each excluded well. In other words, we created 56 × 5 = 280 AIs. For the data from the single well (the data from the single well that was not used to train the AI), the name of the drug was identified based on the five models we created, and the mean value was calculated. The deviation of the five models prediction accuracy was 0.11% at the trained concentrations of all drugs. The deviation of the prediction accuracy at all concentrations of all drugs was 1.6%. The predictive probabilities at different drug concentrations are shown in Table 4. DMSO and acetaminophen, which are seizure-free compounds, were judged to be seizure-free at all concentrations for every drug vehicle, with a mean probability of 99.9% ± 0.3%. 4-AP (1 µM), amoxapine (3 µM), NMDA (1 µM), picrotoxin (0.1 µM), pilocarpine (1 µM), PTZ (10 µM), theophylline (3 µM), varenicline (10 µM), venlafaxine (3 µM), and tramadol (10 µM) were correctly identified at concentrations lower than those in the training data. Carbamazepine (30 µM), kainic acid (1 µM), and paroxetine (3 µM) were correctly identified at the concentrations used to train the AI. The drugs that could not be identified at certain concentrations were all seizure-free compounds, and no drugs were misidentified as different drugs. The mean predictive accuracy for all drugs at the concentrations used to train the AI was 99.9% ± 0.1%. The drug identification AI we created correctly identified the responses of 13 seizure-causing compounds and two seizure-free compounds.

**Table 3:**
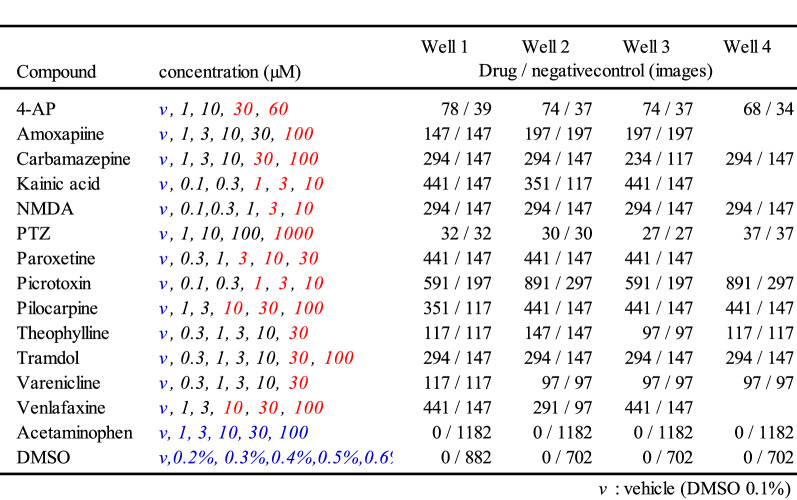
Dataset information used for the creation of drug name prediction AI.

The concentrations in red letters indicate the concentrations used for learning the drug name, and blue letters indicate the concentration learned as negative control.

**Table 4:**
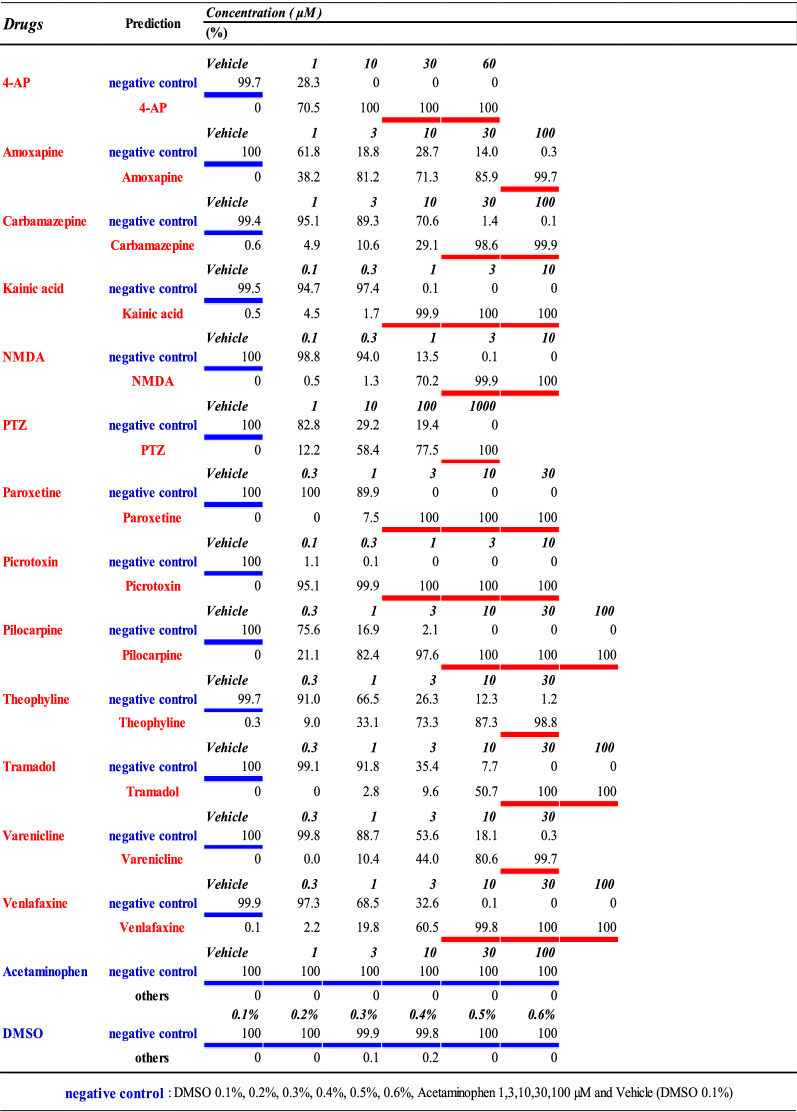
Probability of drug name in each concentration data predicted by AI.

The underlining shows the concentration data used for learning the drug name (red), and the negative control (blue).

## Discussion

There are multiple studies that reported on the evaluation of seizure-causing compounds via MEA measurement using rodent neurons^18,23,35^. However, in recent years, there have also been reports on evaluating seizure-causing compounds using iPS neurons^23^. The results of previous research on in vitro assays^18,23,35,37–42^, the concentrations in the present study at which significant differences were observed using conventional burst analysis, the concentrations at which the AI identified seizure liability, and the concentrations at which the AI identified drugs are all summarized in Table 5. Table 5 shows the concentrations and analysis parameters that were significantly different from those of the vehicle studied in a previous research^18,23,35,37–42^ and the present study using the conventional analysis method. It can be seen that the parameters at which significant differences are detected differ depending on the type of drug, the type of neurons, and the facilities.

**Table 5:** Comparison with pre-clinical seizure assay benchmark using 15 compounds.

Compound	Concentration range tested	Rodent	iPS	iPS (this work)	iPS (this work)	iPS (this work)
		^a^Conventional burst analysis	^a^Conventional burst analysis	^a^Conventional burst analysis	^a^AI	Drug name AI
4-Aminopyridine	1–60 µM	3 µM^23^ (NBP, DIQR)	1 µM^23^ (CV of IBI)	< 1 µM (NoB, IBI, DoB, SiB)	< 1 µM	< 1 µM
Amoxapine	1–100 µM	0.03 µM^23^ (DoB)	0.03 µM^23^ (NEBP)	N/A	N/A	3 µM
Carbamazepine	1–100 µM	0.75 µg/0.2 µL^40^ (in vivo)	–	30 µM (TS, NoB)	30 µM	30 µM
Kainic acid	0.3–10 µM	0.4 µg/0.2 µL^37^ (in vivo)	–	0.3 µM (TS)	1 µM	1 µM
NMDA	0.1–10 µM	5 µM^18^ (TS, NoB, SiB, PIS, ISICV, DoB, IQRBD, IBI, ISId, MBSN, ISI )	–	0.3 µM (TS)	1 µM	1 µM
Paroxetine	0.3–30 µM	5 mg/kg^41^ (in vivo)	–	< 0.3 µM (DoB)	3 µM	3 µM
Picrotoxin	0.1–10 µM	0.3 µM^23^ (ISI)	0.1 µM^23^ (CV of IBI)	0.3 µM (DoB)	< 0.1 µM	< 0.1 µM
Pilocarpine	0.3–100 µM	0.3 µM^23^ (IBI, DoB, SiB)	0.3 µM^23^ (ISI)	10 µM (IBI)	1 µM	1 µM
PTZ	1–1000 µM	100 µM^23^ (TS, NoB)	< 30 µM^23^ (IBI)	< 1 µM (NoB, IBI, SiB)	< 1 µM	10 µM
Theophylline	1–100 µM	300 µM^35^ (in slice)	–	3 µM (IBI)	N/A	3 µM
Tramadol	0.3–100 µM	80.8 mg/kg^39^ (in vivo)	–	3 µM (NoB, SiB)	3 µM	10 µM
Varenicline	0.3–30 µM	0.25 mg/kg^38^ (in vivo)	–	30 µM (IBI, DoB)	10 µM	10 µM
Venalfaxine	0.3–30 µM	25 mg/kg^42^ (in vivo)	–	10 µM (DoB)	3 µM	3 µM
DMSO	0.1–0.6%	N/A^23^	N/A^23^	N/A	N/A	N/A
Acetaminophen	1–100 µM	N/A^23^	N/A^23^	3 µM (DoB)	N/A	N/A
Aspirin	1–100 µM	–	–	3 µM (TS, IBI)	N/A	–
Amoxicilline	1–100 µM	N/A^23^	1 µM^23^ (IBI)	< 1 µM (NoB)	N/A	–
Ferbinac	1–100 µM	–	–	10 µM (SiB)	N/A	–

*NEBP* no. of elecs. participating in burst, *PIS* percent isolated spikes, *ISI* inter spike intervals, *ISI CV* coefficient of variation of the ISIs, *DIQR* interquatrile range of burst duration of burst duration, *MBSN* normalized MAD burst spike number, *ISId* ISI distance, – , no report; *N/A* no risk.

^a^Riskjudge.

A comparison of the results in which compounds were judged to have seizure liability via conventional parameter analysis and predicted to exhibit seizure liability by the AI using the MEA data obtained in the present study showed that aside from theophylline and amoxapine, seizure liability was determined at virtually all concentrations. This result indicated that the AI developed in the present study using raster plots is able at the very least to grasp the characteristics of the parameters obtained from burst analysis. In conventional parameter analysis, the parameters that change differ depending on the drug (Table 5), so it is unclear which parameter should be focused on to best detect toxicity. If a compound is determined to exhibit toxicity when there is a change in just a single parameter, as was the case for acetaminophen, a seizure-free compound may be determined to have seizure liability (a false positive). Even in previous studies, acetaminophen is considered a non-neurotoxic (negative controls) compound, which produced only sporadic changes in the measured parameters^43^. However, the AI developed in the present study judged the seizure liability of acetaminophen, Aspirin, amoxicillin, and felbinac (seizure-free compounds) at all concentrations. This AI prediction method is able to grasp the characteristics of seizure liability, which means that it does not produce false positives. Theophylline has been reported to have seizure liability only at a high concentration (300 µM) based on brain slice responses^35^, so the seizure liability result produced by the AI is valid. In our in vivo rat study, the cerebrospinal fluid (CSF) drug concentration immediately after theophylline administration had seizures was 1570 µM. The CFS concentration was 605 µM when only prodromal symptoms did not cause seizures (data not shown). From these facts, it can be inferred that seizures occur at a higher concentration than the concentration used in this in vitro MEA study. The conventional MEA analysis results judge concentrations of 3 µM to have seizure liability (Fig. 2i), indicating a high probability of false positives. The results of the present study indicate that the seizure liability prediction AI is a method that can reduce the number of false positives compared to that of conventional MEA analysis. Amoxapine was not determined to be at seizure risk. The analysis parameters in Fig. 2 also showed no significant changes, suggesting that the AI determination is valid and is characteristic of the cell model used. Selection of human iPS cell-derived neurons that are reactive to all positive compounds will also be important for improving prediction accuracy.

Additionally, the AI calculates the probability of toxicity risk, which means that it is able to perform concentration-dependent judgments and rank compounds in the order of their seizure liability. Venlafaxine, tramadol, and paroxetine are serotonin reuptake inhibitors that are also seizure-causing compounds; at a concentration of 3 µM, the AI is able to rank them in order of toxicity risk as follows: paroxetine (73.7%), followed by tramadol (61.9%), and venlafaxine (43.3%). For tramadol, there are reports of seizures at 300 mg administration^44^, and at 200 mg administration, the blood concentration is 0.6 µg / mL^45^, and there is a report that there is a linear system in concentration and blood concentration^46^. Therefore, the blood concentration at 300 mg is calculated to be about 3 µM. The toxicity risk of AI at 3 µM was 61.9%. Although it is the blood concentration, the judgment result of tramadol by AI is considered to indicate the possibility of in vitro to in vivo extrapolation (IVIVE).

The concentrations at which the drug identification AI was able to identify drugs were virtually the same as the concentrations at which it was able to identify toxicity risk. This indicates a marked appearance of responses unique to drugs with different mechanisms at concentrations of seizure-causing compounds that result in seizure liability. Additionally, the AI successfully identified amoxapine and theophylline, the two drugs that were judged not to exhibit toxicity. This indicates that even if there was no toxicity risk, the AI was still able to detect the characteristics of the drugs, and it was not necessarily basing its judgments on toxic concentrations or the mechanism of action. These results show that the AI is able to identify the characteristics of each drugs, which in turn means each mechanism of action, even for the same seizure-causing compound. The drug identification AI is able to predict what known compound an unknown compound resembles, and what the mechanism of action is, which holds promise in the field of drug discovery.

One characteristic of the AI that we developed is that it was created using raster plots. There are two benefits to using images that were segmented into windows of time four times the IMFI. The first is that the AI is able to grasp the time-series characteristics outside of simply the mean value of the burst firing or the whole measurement time. The AI determined that pilocarpine was toxic at concentrations lower than the analysis parameters. Looking at the raster plots, the burst cycle of pilocarpine tends to be bunched together. The AI is able to interpret the regularity of the network burst firing and so can determine the toxicity risk at low concentrations more effectively than conventional analysis methods. The second benefit is that there is no difference between the burst firing frequency between samples. As Fig. 1B shows, even in cases in which the firing frequency at the initial state (vehicle) differ depending on the sample, the AI successfully predicted the toxicity and mechanism of the compound. In other words, the difference between samples can be processed by normalizing with the 4096-dimensional features of the vehicle. The method in which the plots are created using the IMFI is considered to be a robust method that can handle inter-well and inter-lot differences. This is also a merit when training the AI on additional drug data.

In this research, we developed AI using features derived from raster plots, but there were problems that involved complicated feature engineering such as standardization of features and that it was difficult to interpret the insights of the judged results. Machine learning methods using simpler spike time series information and burst-related parameters are considered to be effective methods that can solve these problems. We predicted seizure risk using the one class SVM method with spike time series information and the one class SVM method with burst related parameters, respectively (Figure S1 and Figure S2). Then, we verified the difference with raster plot AI. For the spike time series information, a total of 34 parameters were used, in which the average firing frequency and average inter spike interval (ISI) for 600 s were calculated for the entire well and for each of the 16 electrodes. The following five parameters were used for the burst-related parameters: total spikes (TS), number of network bursts (NoB), inter network burst interval (IBI), duration of a network burst (DoB), and spikes in a network burst (SiB). The dataset used for training and testing was the same as the dataset used to create the raster plot AI (Supplementary information). In one class SVM using the spike time series information and burst-related parameters, the risk assessment of the positive and negative compounds of the trained data was achieved, but the untrained data of acetaminophen was judged to be positive. In addition, the concentration-dependence of seizure risk of NMDA, PTZ, and carbamazepine could not be detected (Figs. S1 and S2). Since the SVM that learned the spike time series information and burst-related parameters was able to detect the seizure risk of 14 positive compounds (Figs. S1 and S2), it was considered to have a potential of a seizure risk prediction method. However, since unlearned negative compounds were judged to be positive, it will be necessary to consider the risk of false positives. In addition, since some compounds did not detect concentration dependence, we considered that the toxicity information was insufficient since only the spike time series information and burst-related parameters was used in this verification. Next, a Multi class SVM using time series information and burst-related parameters was created using the leave-one-well-out scheme in the same way as the raster plot AI was created (Supplementary information), and drug name prediction was performed (Tables S6 and S7).

However, of the 14 positive compounds, the time-series information model was able to predict the drug names of only two compounds, and the model that learned the burst-related parameters was able to predict the drug names of only six compounds. Since low-concentration data of positive compounds are judged to be other positive compounds rather than negative compounds (Tables S6 and S7), it is considered that simple time series information and burst-related parameters do not capture the characteristics of each compound. In particular, it is probably difficult to predict compounds with similar parameter changes. Raster plot AI using 4096-dimensional features is considered to have sufficient information to detect compound differences, and compound classification is an advantage of raster plot AI. The AUC results for SVM and NN shown in Fig. 3C were 0.9967 and 0.9841, and the high values were shown even if the models were different, suggesting that it is effective to use the features of the raster plot.

As a future issue, we need to test whether this AI can predict safe neuroactive compounds as negative. In addition, the current limitation of this AI model has been described below. The developed AI does not show high prediction accuracy for various human iPSC sources or rodent neurons with different firing patterns. It is necessary to create a new AI for each human iPSC sources or rodent neurons. Supplementary Fig. S4 shows the results of predicting rodent neuron data using AI prepared from the hiPSC-derived neurons data shown in this study. The toxicity of seizure-causing compounds has not been clearly determined. However, when AI was generated using Rodent neuron data, the four trained seizure-causing compounds (4-AP, PTZ, tramadol, and carbamazepine) and seizure-free compounds (acetaminophen and DMSO) were successfully separated (supplementary Fig. S3). In addition, untrained seizure-causing compounds (picrotoxin, paroxetine, and pilocarpine) were judged to be toxic in a concentration-dependent manner, and unlearned seizure-free appliecompounds (Aspirin and amoxicillin) were judged to be negative in all concentrations. Furthermore, theophylline was judged to be negative, similar to hiPSC-derived neurons. The developed AI is considered to be an effective method for predicting the seizure risk using specific iPSC-derived neurons or rodent neurons in house. In the future, it is necessary to create common AI with high prediction accuracy for various hiPSC sources or rodent neurons.

This study was conducted via cumulative administration. One-well-one-dose studies may be desirable for drugs of concern for desensitization. The raster plot machine learning method can be applied to not only cumulative administration tests but also to one-well-one-dose tests. To improve the accuracy of toxicity detection, selecting the administration test method in consideration of the test compound is important.

An important issue facing in vitro toxicity screening systems is in vitro to in vivo extrapolation (IVIVE). In in vitro MEA measurements, the criteria for determining toxicity have not been clarified even in the conventional analysis method. To determine whether or not the in vivo results are consistent with the findings of the present study, future in vivo studies of the drugs at the concentrations that are known to exhibit toxicity are warranted. Comparing the CSF concentrations of compounds during seizures in animal experiments is considered an effective method of approaching IVIVE. In other words, the CSF concentration during a seizure can be used as teaching data to indicate that a particular compound in the in vitro data represents a seizure-causing compound. This can help create a seizure liability AI that uses CSF concentration during a seizure as a reference. Also, the threshold for judging a compound to be toxic (the threshold of the probability to determine toxicity risk by the AI in one plot) used in the present study was 50% or higher. However, changing the threshold settings for AI toxicity determination may lead to results that are in line with in vivo data. In any case, there is a need to obtain data from animal experiments. However, the current in vitro model can determine whether a compound is a seizure-causing or seizure-free compound and can detect concentration dependence. It is also able to rank compounds in order of their seizure liability. Selecting lead compounds based on seizure liability holds promise in the practical application of this model, as it is considered useful to prioritize the development of new compounds.

The AI for this study was constructed using a CNN model. In general, recurrent neural networks (RNNs) are often used for deep learning of the concept of time. However, in in vitro MEA data, the temporal change of the firing pattern within a certain concentration is not observed so much, and the concentration dependence is more remarkable. In addition, the frequency of synchronized burst firing before drug administration differs in vitro, and we divide the raster plot image by the number of network bursts to accommodate the time-scale differences in neural activity between samples. In summary, optimized raster plot images were created to extract the characteristics of dose-dependent neural activity independent of sample differences. A CNN model was adopted to extract the features of neural activity from a single raster plot image.

The developed AI also used AlexNet, which is not specialized for learned tasks and can be applied to a wide range of tasks. After extracting the 4096-dimensional features, seizure-risk was determined using a neural network, which is machine learning. Models are being developed daily; hence, finding a more optimal model should be considered as the next issue.

Predicting seizure liability using AI holds promise as a method of toxicity screening that is able to use standardized indicators, which would improve upon the current toxicity screening methods that use different standards (parameters) depending on the institution conducting the research. It requires further study, but because the method described in the present study uses the IMFI and is not influenced by the initial state of the sample, this model may be able to make predictions at different institutions using cells from different vendors. It is also possible that if the AI is able to identify the basis for discerning between compounds using AI, this model may hold promise in the dissemination of neurological knowledge and the discovery of more detailed mechanisms of actions of compounds, as the drug identification AI is able to distinguish even between seizure-causing compounds with the same mechanism.

The present study showed that analysis by the AI we developed using raster plots is effective as a means of predicting seizure liability and the mechanism of action of compounds. This suggests that this model is able to detect concentration dependence in seizure liability that cannot be detected using single parameter analysis and also to distinguish detailed differences between compounds even when their main effects are the same. This model has a high precision in predicting the seizure liability and mechanism of unknown compounds based on in vitro MEA data and may become a new analysis method that can be used uniformly across all institutions.

## Materials and methods

### Culture of human iPSC-derived cortical neurons

Human iPSC-derived cortical neurons (XCL-1,XCell Science Inc., USA) were cultured at a concentration of 3.0 × 10^5^ cells/cm^2^ (16 channels per well) across 24-well MEA plates (Comfort; Alpha Med Scientific) coated with polyethyleneimine (Sigma) and laminin-511 (Nippi). For culture on MEA, a ϕ3.4-mm glass ring was placed in the middle of the MEA probe at the location of the electrode array, and cell suspensions were seeded inside the ring. After 1 h, neural maturation basal medium (NM-001-BM100, XCell Science Inc., USA) supplemented with neuron maturation supplement A (NM-001-SA100, XCell Science Inc., USA) and 100 U/mL penicillin/streptomycin (168-23191, Wako) was applied around the ring, and the ring was carefully removed. After 8 days of culture, the medium was replaced with BrainPhys neuronal medium with SM 1 neuronal supplement (STEMCELL technologies, USA). After medium exchange on 8 days of culture, human iPSC-derived mature astrocytes (XCL-1 mature astrocytes, AR-001-1V, XCell Science) were seeded at 3.0 × 10^4^ cells per well where human iPSC-derived neurons were already cultured. Half the media was exchanged every 4 days.

### Rat primary cortical neurons culture

The animal experiments were performed with the approval of the Animal Experiment Ethics Committee at the Tohoku Institute of Technology (approval number: 589) and according to the Tohoku Institute of Technology guidelines for the Care and Use of Laboratory Animals and the ARRIVE guidelines. Rats were housed under standard laboratory conditions (12-h light/12-h dark cycle, free access to food and water). These experimental protocols were conducted in accordance with the Fundamental Guidelines for Proper Conduct of Animal Experiment and Related Activities in Academic Research Institutions (Ministry of Education, Culture, Sports, Science and Technology, Notice No. 71 of 2006), the Standards for Breeding and Housing of and Pain Alleviation for Experimental Animals (Ministry of the Environment, Notice No. 88 of 2006), and the Guidelines on the Method of Animal Disposal (Prime Minister's Office, Notice No. 40 of 1995). MED Multi-well probe (MED-R5NF30; Alpha MED Scientific Inc, Osaka, Japan) with 16 electrodes in each well were used for this experiment. One day before seeding, MEA probes were coated with 0.1% polyethyleneimine dissolved in a borate buffer at 37 °C, 5%CO_2_ for 1 h. After 4 times washing, the probes were allowed to air dry overnight in a safety cabinet. About 2 h before seeding, the electrodes in a probe were covered with 5µL of 20 µg/mL i-Matrix (Nippi, Tokyo, Japan) solution and incubated in 37 °C, 5% CO_2_ condition. Cortical neurons gathered from brains of rat fetus (Charles River, Yokohama, Japan) at GD17-18 were prepared through 0.25% trypsin (ThermoFisher, MA, USA) and DNase (Roche Diagnostics K.K., Tokyo, Japan) treatments. After removing i-Matrix solution, cell suspension (5.0 × 10^4^ cells/5 µL) was immediately dropped in the electrode area in the probes, and the probes were incubated at 37 °C and 5% CO_2_ for 1 h before adding 1 mL medium per well. The day after seeding the cortical neuron, all medium was replaced freshly. Neurobasal medium (ThermoFisher, MA, USA) with 1.8% B-27 supplement (ThermoFisher, MA, USA), 0.9% Glutamax (ThermoFisher, MA, USA), 0.9% penicillin/streptomycin (ThermoFisher, MA, USA), and 10% neuron culture medium (FUJIFILM Wako Pure Chemical Corporation, Osaka, Japan) was chosen as culture medium in this study. The probes were cultured in the incubator at 37 °C and 5% CO_2_ and half volume (500 µL) of culture medium in each well were exchanged twice a week. One day before the MEA recording, all culture medium was changed for measurement.

### Extracellular recording

Spontaneous extracellular field potentials were acquired at 37 °C under a 5% CO_2_ atmosphere using a 24-well MEA system (Presto; Alpha Med Scientific) at a sampling rate of 20 kHz/channel. The spikes in the acquired data were detected using a 100-Hz high-pass filter. The measurement time was controlled by Presto software (Alpha Med Scientific), and all the raw data was saved on the PC.

### Pharmacological tests

After 14th weeks of culture, 13 convulsant compounds and 2 negative compounds were cumulatively administered to induce seizure-like events in human iPSC-derived cortical neurons. The following compounds were used as convulsants: the potassium channel blocker, 4-aminopyridine (4-AP; 1, 10, 30, 60 µM: 275875-1G, Sigma-Aldrich), the GABAA receptor antagonist, picrotoxin (0.1, 0.3, 1, 3, 10 µM: P1675-1G, Sigma–Aldrich), 1,5-pentamethylene-tetrazole (PTZ; 1, 10, 100, 1000 µM: P0046, Tokyo Chemical Industry Co.), the sodium channel blocker, carbamazepine (1, 3, 10, 30, 100 µM: C4024-1G, Sigma-Aldrich), the muscarinic ACh receptor agonist, pilocarpine (0.3, 1, 3, 10, 30, 100 µM: P6503-5G, Sigma-Aldrich), the centrally acting synthetic opioid analgesic and norepinephrine and serotonin reuptake inhibitor, tramadol (0.3, 1, 3, 10, 30, 100 µM: 42965-1G, Sigma-Aldrich), the serotonin uptake inhibitor, paroxetine (0.3, 1, 3, 10, 30 µM: PHR1804, Sigma-Aldrich), the norepinephrine and serotonin reuptake blocker, venlafaxine (0.3, 1, 3, 10, 30 µM: PHR1736-1G), the norepinephrine and serotonin reuptake and dopamine receptor blocker, amoxapine (1, 3, 10, 30, 100 µM: A129-100MG, Sigma-Aldrich), the adenosine receptor antagonist, theophylline (1, 3, 10, 30, 100 µM: T1633-50G, Sigma–Aldrich), kainic acid (0.1, 0.3, 1, 3, 10 µM: K0250-10MG, Sigma-Aldrich), N-methyl-D-aspartic acid (NMDA; 0.1, 0.3, 1, 3, 10 µM; M3262-25MG、Sigma–Aldrich), and the α4β2 nicotinic acetylcholine receptor agonist, varenicline (0.3, 1, 3, 10, 30 µM: PZ0004-5MG, Sigma-Aldrich). COX-2 inhibitor was used to treat pain and fever. Acetaminophen (1, 3, 10, 30, 100 µM: A7085-100G, Sigma-Aldrich) , Aspirin (1, 3, 10, 30, 100 µM: A2093-100G, Sigma-Aldrich), Amoxicillin (1, 3, 10, 30, 100 µM: A2099, Tokyo Chemical Industry), 4-Biphenylacetic Acid (Felbinac; 1, 3, 10, 30, 100 µM: B1278, Tokyo Chemical Industry) and the organosulfur solvent, DMSO (0.1%, 0.2%, 0.3%, 0.4%, 0.5%, 0.6%: D2650-5X10ML, Sigma–Aldrich) were used as negative control compounds. The final concentrations of these drugs were adjusted to contain 0.1% DMSO. DMSO (0.1%) was administered in all wells as a vehicle control prior to cumulative administration of the compound. Spontaneous firing was recorded for 10 min at each concentration (n ≥ 3).

### Burst analysis

Electrophysiological activity was first analyzed using Presto and Mobius software (Alpha Med Scientific) and MATLAB. A spike was counted when the extracellularly recorded signal exceeded a threshold of ± 5.3 σ, where σ is the standard deviation of the baseline noise during quiescent periods. Network bursts were detected using the four-step method^33^.

### Statistical analysis

To determine the seizure liability, one-way ANOVA followed by Dunnett's test were used to calculate the significant difference between each concentration and the vehicle. The concentration showing a significant difference in the parameters calculated by the burst analysis was determined as the risk concentration.

### Predictive model performance evaluation

To validate the performance of the models, we plotted the receiver operating characteristic (ROC) curve and then calculated the area under the ROC curve (AUC). The ROC curve provides a means of comparison between classification models. Additionally, the ROC curve shows the false positive rate on the X-axis against the true positive rate on the Y-axis. The ROC curve is a plot of false-positive and true-positive probabilities for all datasets when the threshold is varied from 0 to 1 for the positive probabilities calculated for a single image. Ideally, the curve will climb steeply toward the top-left, meaning that the model gives correct predictions. The AUC is a measure of the quality of the classification models. A random classifier has an AUC of 0.5, while a perfect classifier has an AUC equal to 1.

Next, to obtain the optimal operating point for the ROC curve, we determined the slope (S) using the following calculation:

$$\mathrm{S}= \frac{Cost\left(P|N\right)-Cost\left(N|N\right)}{Cost\left(N|P\right)-Cost\left(P|P\right)}* \frac{N}{P}$$

where Cost(N|P) is the cost of misclassifying a positive class as a negative class. Cost(P|N) is the cost of misclassifying a negative class as a positive class. P and N are the total instance counts in the positive and negative classes, respectively. Next, we determined the optimal operating point by moving the straight line with slope S from the upper left corner of the ROC plot (FPR = 0, TPR = 1) down and to the right, until it intersected the ROC curve.

The statistical tests used to validate the performance of the models using the optimum operating point were accuracy, positive predictive value (PPV), sensitivity, specificity, and the F-measure. These metrics are prevalent in the machine learning community for classification tasks. Accuracy is a measure of the number of the sum of true positives and true negatives divided by the total number of datasets. PPV is a measure of the number of true positives divided by the sum of the true positives and the false positives. Sensitivity is a measure of the number of true positives divided by the sum of the true positives and the false negatives. Specificity is a measure of the number of true negatives divided by the sum of true negatives and false positives. The F-measure represents the balance that exists between the precision and recall scores. The F-measure is defined as the product of precision and recall divided by the sum of precision and recall.

### Support vector machine (SVM) model

The raster plot images were converted to feature vectors using the Alexnet model. The 21st layer of Alexnet, called fc7, wherein each image was represented in a 4096-dimensional vector, was used for input features in SVM. The feature datasets were separated using linear SVM. Hold out validation was used to verify the data separation, and the AUC of the ROC curve was calculated to evaluate the accuracy of the prediction. Our algorithms were developed using MATLAB’s Statistics and Machine Learning Toolbox. For machine learning-based classification, SVM-based supervised learning was performed using the “fitcsvm” and “predict” functions from MATLAB’s Statistics and Machine Learning Toolbox.

## Supplementary Information

Supplementary Information.
