# Supplementary material for: Raster plots machine learning to predict the seizure liability of drugs and to identify drugs
Source: Sci Rep. 2022 Feb 10;12:2281. doi: 10.1038/s41598-022-05697-8 (PMC8831568; doi:10.1038/s41598-022-05697-8)
Supplement: Supplementary file 1 — Supplementary Information. [file 41598_2022_5697_MOESM1_ESM.docx]

Supplementary information

Raster plots machine learning to predict the seizure liability of drugs and to identify drugs

N. Matsuda,^1^ A. Odawara,^1^ K. Kinoshita,^2^ A. Okamura,^2^ T. Shirakawa,^2^ I. Suzuki^1^*

^1^Department of Electronics, Graduate School of Engineering, Tohoku Institute of Technology, 35-1 Yagiyama Kasumicho, Taihaku-ku, Sendai, Miyagi, 982-8577, Japan

^2^ Drug Safety Research Labs, Astellas Pharma Inc. 21 Miyukigaoka, Tsukuba, Ibaraki, 305-0841, Japan

**^*^Corresponding author:**

Ikuro Suzuki

Tel: +81-22-305-3219

Fax: +81-22-305-3219

E-mail: [i-suzuki@tohtech.ac.jp](mailto:i-suzuki@tohtech.ac.jp)

**Supplementary Methods**

**Extracellular recording in rat primary cortical neurons**

The extracellular signals in spontaneous activity were recorded by using the MEA systems (MED Quad II; Alpha Med Scientific). An input range 2.3 mV and low cut filter of 0.1 Hz and high cut filter of 10000 was used. MEA probes were maintained at 37 °C in a 5% CO_2_ incubator during the recording. Spikes and bursts recording were performed using Mobius software (Alpha Med Scientific). To avoid the difference of probe, a cumulative method was applied in this study. Prior to treatment of compounds or vehicle, the MEA probe was set in a chamber which were maintained at 37 °C in a 5% CO_2_, for at least 10 minutes. Firstly, 0.1% DMSO dissolved in the culture medium was applied into each well as a vehicle control, and recorded the spontaneous activity for 10 min after gently pipetting. After 10 min recording, the low concentration of the compound was added into each well and spontaneous activity was recorded as same as vehicle control. The middle and high concentration of the compounds were treated repeatedly. The spikes in the acquired data were detected using a 100-Hz high-pass filter.

**Pharmacological tests**

8 convulsant compounds and 4 negative compounds were cumulatively administered to induce seizure-like events in rat primary cortical neurons. The following compounds were used as convulsants: the potassium channel blocker, 4-aminopyridine (4-AP; 0.3, 3, 30 µM: Sigma-Aldrich), the GABAA receptor antagonist, picrotoxin (0.1, 1, 10 µM: Sigma–Aldrich), pentamethylenetetrazole (PTZ; 10, 100, 1000 µM: Sigma-Aldrich), the sodium channel blocker, carbamazepine (0.1, 1, 10 µM: Sigma-Aldrich), the muscarinic ACh receptor agonist, pilocarpine (0.3, 3, 30, 100 µM: Fujifilm Wako), the centrally acting synthetic opioid analgesic and norepinephrine and serotonin reuptake inhibitor, tramadol (0.3, 3, 30 µM: Sigma-Aldrich), the serotonin uptake inhibitor, paroxetine (0.1, 1, 10 µM: Tokyo chemical Industry), the adenosine receptor antagonist, theophylline (2, 20, 200 µM: Sigma–Aldrich). COX-2 inhibitor was used to treat pain and fever. Acetaminophen (1, 10, 100 µM: Sigma-Aldrich), Aspirin (1, 10, 100 µM: Sigma-Aldrich) and the organosulfur solvent, DMSO (0.1%, 0.2%, 0.3%, 0.4%: Fujifilm Wako) were used as negative control compounds. The final concentrations of these drugs were adjusted to contain 0.1% DMSO. DMSO (0.1%) was administered in all wells as a vehicle control prior to cumulative administration of the compound. Spontaneous firing was recorded for 10 min at each concentration (n ≥ 4).

**Creating an artificial intelligence using raster plot**

8 seizure-causing compounds and 4 seizure-free compounds datasets, which is the number of split raster plots per concentration, were 4-AP (n = 4 wells): 220 images / concentration, PTZ (n = 4 wells): 579 images / concentration, Tramadol (n = 4 wells): 520 images / concentration, Carbamazepine (n = 4 wells): 432 images / concentration, Acetaminophen (n = 4 wells): 186 images / concentration, DMSO (n = 8 wells): 648 images / concentration, Picrotoxin (n = 4 wells): 696 images / concentration, Paroxetine (n = 4 wells): 676 images / concentration, Pilocarpine (n = 4 wells): 326 images / concentration, Theophylline (n = 4 wells): 608 images / concentration, Aspirin (n = 4 wells): 353 images / concentration, and Amoxicillin (n = 4): 356 images / concentration. We used four seizure-causing compounds with different mechanisms and burst frequency responses (4-AP [30, n = 3 wells], tramadol [30 µM, n = 3 wells], PTZ [1000 µM, n = 3 wells], and carbamazepine [10 µM, n = 3 wells]) and two seizure-free compounds (all concentrations of acetaminophen [n = 3 wells] and all concentrations of DMSO [n = 3 wells]) to train and validate the effectiveness of this model; 75% of the dataset was used for training, and the remaining 25% was used for validation after training. The accuracy was evaluated using the raster plots of unlearned wells after training. The training data used contained 191 4-AP plots, 524 PTZ plots, 394 tramadol plots, 380 carbamazepine plots, 116 acetaminophen plot, 490 DMSO plots, and 1489vehicle plots.

**Creating a one class SVM**

In order to compare the features of the raster plot with the trained AI, we created a one class SVM that trained the spike time series information and burst-related parameters. For the spike time series information, a total of 34 parameters were used, in which the average firing frequency and average inter spike interval (ISI) for 600 seconds were calculated for the entire well and for each 16 electrodes. The following five parameters were used for the burst-related parameters: total spikes (TS), number of network bursts (NoB), inter network burst interval (IBI), duration of a network burst (DoB), and spikes in a network burst (SiB). All concentrations of the negative compounds acetaminophen (n = 3 well) and DMSO (n = 3 well), and the solvent data (vehicle) of the positive compounds 4-AP (n = 3 well) and carbamazepine (n = 3 well), NMDA (n = 3 well) and PTZ (n = 3 well) were used for learning and testing. The wells used for training and test data are similar to the wells used to create the raster plot AI. Our algorithms were developed using MATLAB’s Statistics and Machine Learning Toolbox. For machine learning-based classification, SVM-based supervised learning was performed using the “fitcsvm” and “predict” functions from MATLAB’s Statistics and Machine Learning Toolbox.

**Creating a multi class SVM**

For comparison with drug name prediction AI, we created a multi class SVM that learned spike time series information and burst related parameters. For the time series information and burst-related parameters, the same parameters as for one class SVM were used. The model was trained on a dataset composed of 4-AP, amoxapine, carbamazepine, kainic acid , NMDA, PTZ, paroxetine, picrotoxin, pilocarpine, theophylline, tramadol, varenicline, and venlafaxine as well as all concentrations of acetaminophen as well as all concentrations of DMSO as seizure-free compounds. The all compounds dataset that was used was made up of 56 wells. Training was conducted by excluding one of the 56 wells. The excluded well was used for obtaining test data. The prediction accuracy was calculated using the leave-one-sample (well)-out scheme. Our algorithms were developed using MATLAB’s Statistics and Machine Learning Toolbox. For machine learning-based classification, SVM-based supervised learning was performed using the “fitcecoc” and “predict” functions from MATLAB’s Statistics and Machine Learning Toolbox.

**Supplementary Discussion S1**

**Seizure liability prediction by one class SVM using spike time series information and burst related parameters**

Supplementary figure S1 shows the anomaly score at each concentration of the compound output by the SVM that learned the time series information. The anomaly score indicates the distance from the boundary of the outliers (p ≤ 0.05) drawn in the feature space, which is calculated from the trained negative compounds and vehicle data, and the negative score indicates the outliers. The prepared time-series SVM detected abnormalities of positive compounds with 100% accuracy in the learned wells, and did not detect abnormalities of negative compounds at all concentrations (Fig. S1A). However, untrained wells with acetaminophen 1, 3, 10, 30, and 100 µM and DMSO 0.2%, 0.3%, 0.4%, 0.5%, and 0.6%, respectively, were determined to be abnormal (Fig. S1A). In the untrained data, the positive compound, Carbamazepine 10 µM, was determined to be normal. Furthermore, no concentration-dependence was found in the anomaly scores of NMDA, PTZ, and Carbamazepine in the unlearned data. (Fig. S1A). Supplementary figure S1B shows the anomaly score at each concentration of unlearned compound. All 9 unlearned positive compounds were determined to be abnormal, and the increase in anomaly scores of 7 positive compounds except Amoxapine and Theophylline was concentration-dependent.

Supplementary figure S2 shows the anomaly score at each concentration of the compound output by the SVM that learned the burst-related parameters. The prepared parameter SVM detected abnormalities in positive compounds with 100% accuracy in the learned wells and did not detect abnormalities in negative compounds at all concentrations. However, unlearned wells of Acetaminophen 10, 30, and 100 µM were judged to be abnormal. In addition, the positive compound NMDA 1 µM was judged to be normal, and concentration-dependent anomalies of NMDA and carbamazepine could not be detected (Fig. S2A). Supplementary figure S2B shows the anomaly score at each concentration of unlearned compound. Eight positive compounds except Amoxapine were determined to be abnormal. Since the burst disappeared after Kainic acid 1 µM, the parameters were not calculated and no score was recorded.

SVMs that learned time series information and burst-related parameters were able to detect the seizure liability of 14 positive compounds. In particular, machine learning based on burst-related parameters is considered to be effective as a method for predicting the seizure liability. However, unlearned negative compounds are judged to be positive, and it will be necessary to consider the risk of false positives. In addition, since some compounds did not detect concentration dependence, it is considered that there is a drug response that cannot be detected by one class SVM using time series information and burst-related parameters.

**Drug name prediction by multi-class SVM using spike time series information and burst related parameters**

Supplementary Table S6 shows the drug name probabilities at each concentration of the compound predicted by the SVM that learned the time series information. Vehicles of all compounds were judged to be negative control. NMDA 0.3 µM and Venlafaxine 10, 30 µM were the only compounds that showed a prediction accuracy of 50% or more, and drug names could not be predicted for other compounds (Table S6). Supplementary Table S7 shows the drug name probabilities at each concentration of compound predicted by SVMs that learned the five burst parameters. Vehicles of all compounds were judged to be negative controls. Compounds showing a prediction accuracy of 50% or more are 4-AP 1, 10, 30, and 60 µM; Carbamazepine 30 and 100 µM; Paroxetine 1, 3, 10, and 30 µM; PTX 0.3, 1, 3, 10 µM; PTZ 10, 100, 1000 µM; and Varenicline 30 µM. The drug name could not be predicted for other compounds and doses. In summary, of the 14 positive compounds, the time-series information model could predict drug names for only 2 compounds, and the model that learned burst-related parameters could predict drug names for only 6 compounds. The low-concentration data of positive compounds are determined to be other positive compounds rather than negative compounds, and it is considered that simple time series information and burst-related parameters do not capture the characteristics of each compound. In particular, it will be difficult to predict compounds with similar parameter changes. Raster plot AI using 4096-dimensional features is considered to have sufficient information to detect compound differences. With more burst-related parameters, prediction accuracy may be improved, but probably not as good as raster plot AI.

**Supplementary Discussion S2**

**Rat primary neural network drug response**

Supplementary figure S3A shows the drug response of each parameter when the vehicle response is set to 100%. The DoB increase in at 30 µM of 4-AP. For PTZ, the TS increased starting at 100 µM, the DoB and SiB increased at 1000 µM. For tramadol, the DoB increased starting at 3µM. For carbamazepine the NoB increased and the IBI and DoB decreased at 10 µM. For DMSO the TS increased at 0.4%. For picrotoxin, the TS increased starting at 0.1 µM and the SiB increased at the 10 µM. For Paroxetine, the TS increased starting at 1 µM and the DoB increased at 10 µM. For pilocarpine, the NoB and SiB increased and the IBI decreased starting at 3 µM and the DoB increased at 30 µM. For theophylline, the NoB increased and the IBI decreased starting at 20 µM. For Acetaminophen, Aspirin and amoxicillin, no significant changes in any parameters were observed.

**Determining concentration-dependent seizure toxicity using the AI which learning rodent data**

Supplementary figure S3B shows the concentration data toxicity probabilities predicted by the AI. For unlearned samples, in which is the data of the wells not used for training dataset, the following concentrations were determined to have a seizure liability probability of 50% or higher —4-AP: 30 µM (100%); PTZ: 1000 µM (100%); Tramadol: 30 µM (63.5%); and Carbamazepine: 10 µM (100%). Acetaminophen, which is a seizure-free compound, was determined to be seizure-free with a probability of 95.7% or higher, regardless of the concentration. DMSO was also determined to be seizure-free with a probability of 98.1% or higher, regardless of the concentration.

**Determining the seizure toxicity of unlearned drugs using the AI which learning rodent data**

Supplementary figure S3C shows the seizure toxicity determination results for each concentration of the unlearned drugs. The concentrations that showed a 50% or higher probability of seizure liability were as follows—Picrotoxin: 1 µM (50.4%) and 10 µM (76.6%); paroxetine: 10 µM (65.3%); pilocarpine: 3 µM (65.3%). Theophylline was determined to be seizure-free at all concentrations, consistent with iPS cell results. Aspirin, which is a seizure-free compound, was determined to be seizure-free with a probability of 72.0% or higher, regardless of the concentration. Amoxicillin was also determined to be seizure-free with a probability of 72.1% or higher, regardless of the concentration.

**Prediction seizure liability of drugs in rat cortical neurons using AI which learned iPSC data.**

Supplementary figure S4 shows the seizure toxicity determination results for concentration of the 12 drugs. The concentrations that showed a 50% or higher probability of seizure liability were as follows—4-AP: 3 µM (50.0%); PTZ: 100µM (55.0%), 1000 µM (64.1%); tramadol: 30 µM (52.1%); pilocarpine: 0.3 µM (53.0%); aspirin: 1 µM (51.4%). Carbamazepine, Picrotoxin, paroxetine and Theophylline were determined to be seizure-free at all concentrations. Acetaminophen, DMSO and Amoxicillin were determined to be seizure-free with a probability of 68.0% or higher, regardless of the concentration.

**
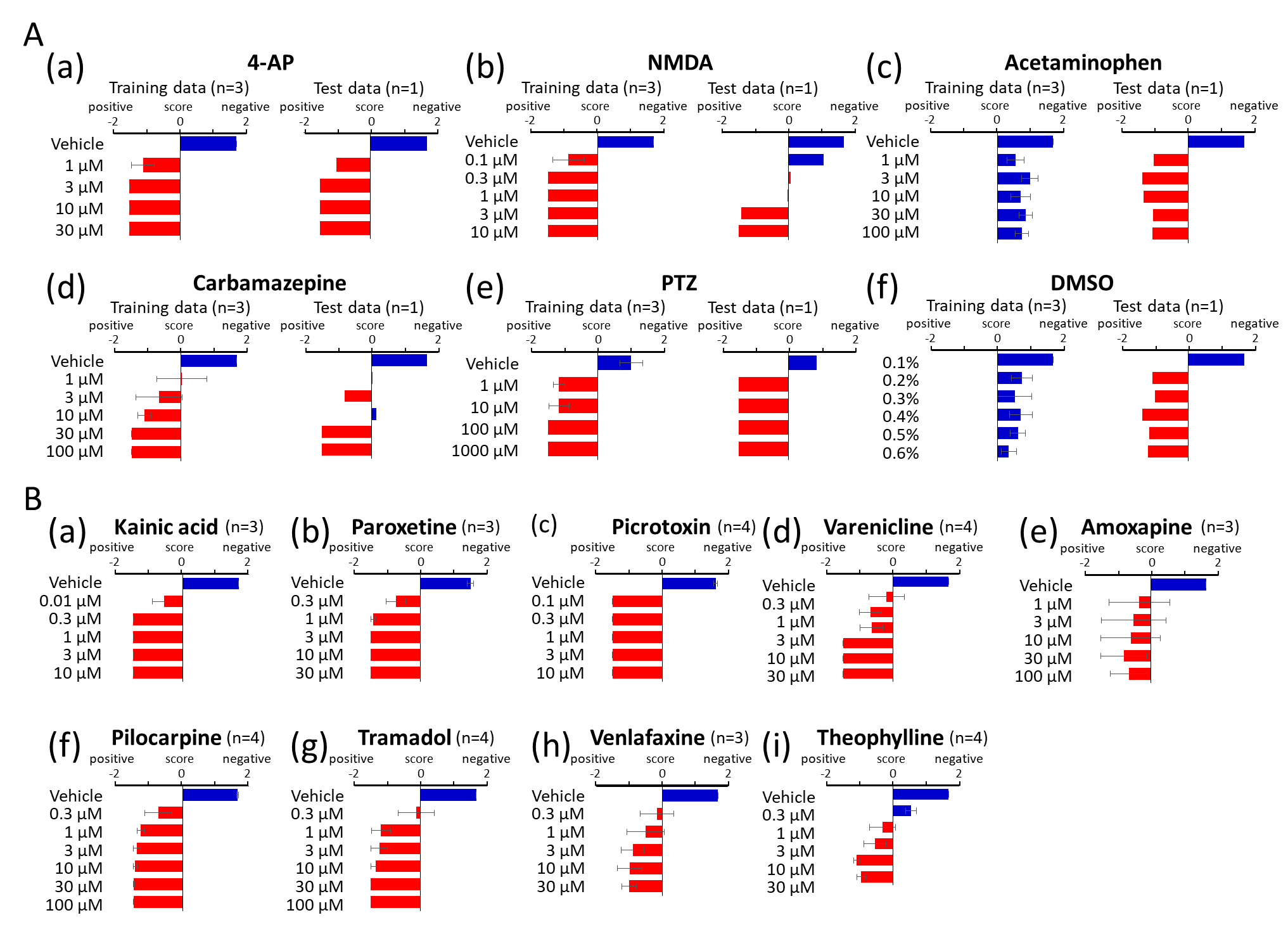
**

**Supplementary Figure S1. Prediction anomaly score of drugs in iPSC data using one class SVM which learned time series data.** Prediction of anomaly score in (A) learning drugs of training data (left) and test data (right). (a) 4-AP, (b) NMDA, (c) acetaminophen, (d) carbamazepine, (e) PTZ, and (f) DMSO. (B) non-training drugs by SVM. (a) Kainic acid, (b) paroxetine, (c) picrotoxin, (d) varenicline, (e) amoxapine, (f) pilocarpine, (g) tramadol, (h) venlafaxine, and (i) theophylline. SVM predicted the negative score (blue bar) and anomaly score (red bar) at each concentration.


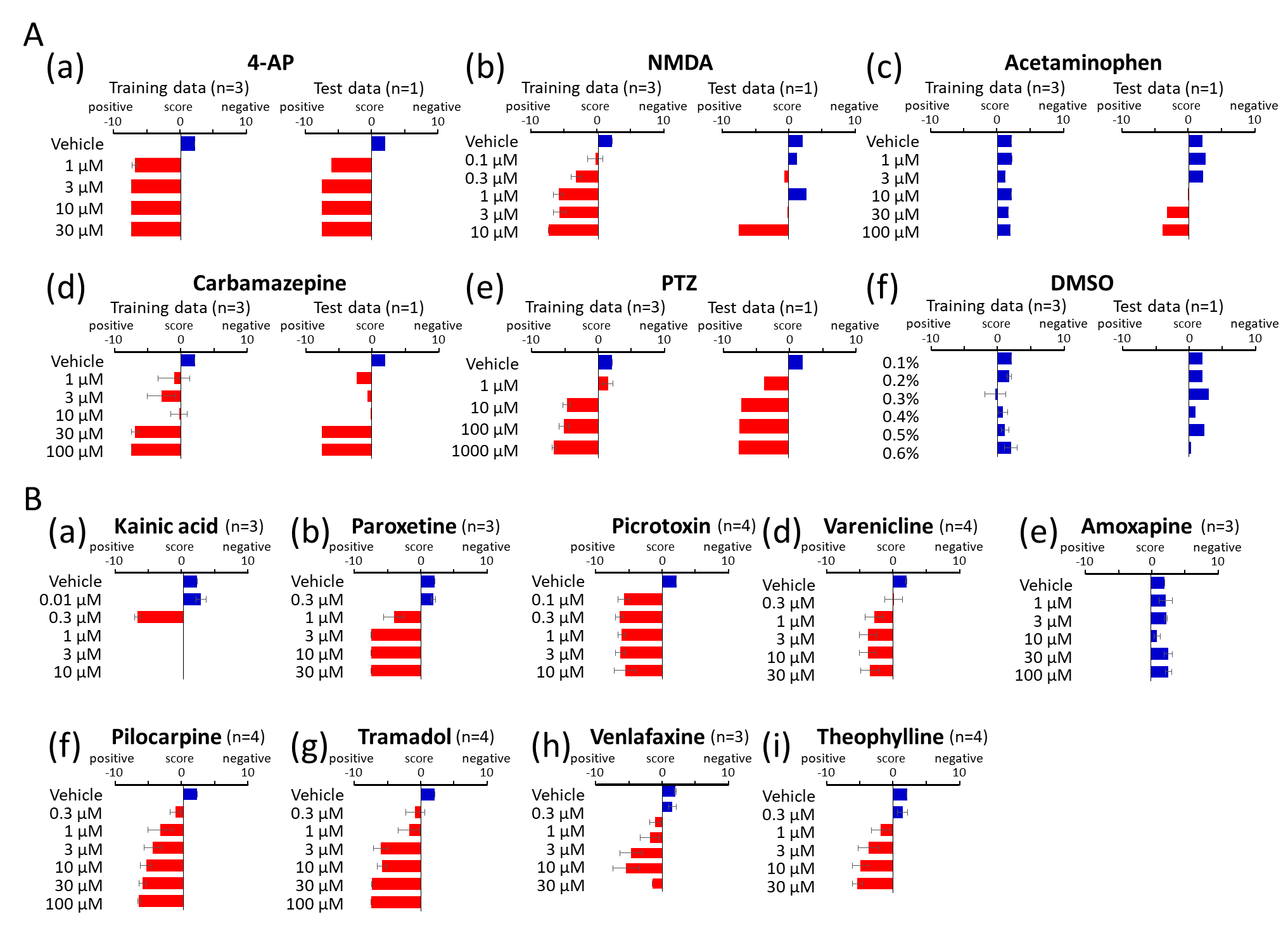


**Supplementary Figure S2. Prediction anomaly score of drugs in iPSC data using one class SVM which learned burst parameters.** Prediction of anomaly score in (A) learning drugs of training data (left) and test data (right). (a) 4-AP, (b) NMDA, (c) acetaminophen, (d) carbamazepine, (e) PTZ, and (f) DMSO. (B) non-training drugs by SVM. (a) Kainic acid, (b) paroxetine, (c) picrotoxin, (d) varenicline, (e) amoxapine, (f) pilocarpine, (g) tramadol, (h) venlafaxine, and (i) theophylline. SVM predicted the negative score (blue bar) and anomaly score (red bar) at each concentration.

**Supplementary Figure S3. Prediction seizure liability of drugs in rat cortical neuron.**

(A) Dose-dependent changes of 12 compounds in five parameters: TS (pink), NoB (black), IBI (green), DoB (blue), SiB (cyan). Parameters were depicted as the average % change of control (vehicle control set to 100%) ± SEM from n = 4-8 wells. Data were analyzed using one-way ANOVA followed by post hoc Dunnett's test (*p < 0.05，**p < 0.01 vs. vehicle).(B) Dose-dependent prediction of seizure risk in learning drugs by AI. AI predicted the negative probabilities (blue bar) and seizure risk (red bar) at each concentration of training data (left) and test data (right). (C) Dose-dependent prediction of seizure risk in non-training drugs by AI. AI predicted the negative probabilities (blue bar) and seizure risk (red bar) at each concentration.

**Supplementary Figure S4. Prediction seizure liability of drugs in rat cortical neurons using AI which learned iPSC data.** Prediction of seizure risk in 12 drugs by AI. AI predicted the negative probabilities (blue bar) and seizure risk (red bar) at each concentration.

**Supplementary Table S1. Concentration-responses of TS in hiPSC-derived neuron.**

**Supplementary Table S2. Concentration-responses of NoB in hiPSC-derived neuron.**

**Supplementary Table S3. Concentration-responses of IBI in hiPSC-derived neuron.**

**Supplementary Table S4. Concentration-responses of DoB in hiPSC-derived neuron.**

**Supplementary Table S5. Concentration-responses of SiB in hiPSC-derived neuron.**


**Supplementary Table S6. Probability of drug name in each concentration data predicted by learned time series SVM.**

**Supplementary Table S7. Probability of drug name in each concentration data predicted by learned burst parameter SVM.**
